# From adjacent activation in *Escherichia coli* and DNA cyclization to eukaryotic enhancers: the elements of a puzzle

**DOI:** 10.3389/fgene.2014.00371

**Published:** 2014-11-03

**Authors:** Michèle Amouyal

**Affiliations:** Interactions à Distance, Centre National de la Recherche ScientifiqueParis, France

**Keywords:** CAP, auto-repression, elongation stop, inducibility, overproduction, cancer therapy, resistance to antibiotics, recombinant DNA

## Abstract

Deoxyribonucleic acid cyclization, *Escherichia coli* lac repressor binding to two spaced lac operators and repression enhancement can be successfully used for a better understanding of the conditions required for interaction between eukaryotic enhancers and the machinery of transcription initiation. Chronologically, the DNA looping model has first accounted for the properties initially defining enhancers, i.e., independence of action with distance or orientation with respect to the start of transcription. It has also predicted enhancer activity or its disruption at short distance (site orientation, alignment between promoter and enhancer sites), with high-order complexes of protein, or with transcription factor concentrations close or different from the wild-type situation. In another step, histones have been introduced into the model to further adapt it to eukaryotes. They in fact favor DNA cyclization *in vitro*. The resulting DNA compaction might explain the difference counted in base pairs in the distance of action between eukaryotic transcription enhancers and prokaryotic repression enhancers. The lac looping system provides a potential tool for analysis of this discrepancy and of chromatin state directly *in situ*. Furthermore, as predicted by the model, the contribution of operators O2 and O3 to repression of the lac operon clearly depends on the lac repressor level in the cell and is prevented in strains overproducing lac repressor. By extension, gene regulation especially that linked to cell fate, should also depend on transcription factor levels, providing a potential tool for cellular therapy. In parallel, a new function of the O1–O3 loop completes the picture of lac repression. The O1–O3 loop would at the same time ensure high efficiency of repression, inducibility through the low-affinity sites and limitation of the level of repressor through self-repression of the lac repressor. Last, the DNA looping model can be successfully adapted to the enhancer auxiliary elements known as insulators.

Any book of molecular biology teaches how eukaryotic genes are activated from regions which are distinct from their genomic localization, sometimes as far as several tens of thousands of base pairs. Specialists of eukaryotic gene regulation often discard or forget that *Escherichia coli* studies have paved the way to the eukaryotic ones (see for example Schleif, 2003 or Amouyal, 2007a). This synthesis presents the initial DNA cyclization hypothesis on which DNA looping for gene regulation and enhancer action relies for most part and the resulting DNA looping model built on the genetic elements of the *E. coli* lac system of repression. It introduces its past and present developments with potential applications.

## FIRST MODELS AND DISCOVERIES (1960–1984)

The more the organism is complex, the more gene expression is controlled by varying conditions and situations. In eukaryotes, genetic expression is often conditioned by a specific stage of development, resulting in pathogenic disorders when the process does not function correctly. These regulations generally involve genomic elements which are both located close and at some distance from the gene. Prokaryotes which are unicellular, do not know cellular differentiation. This is why their genes are generally unbridled. They will only be submitted to similar patterns of genetic expression when they must adapt like eukaryotes to varying environmental and nutritional conditions.

### PROXIMAL ACTIVATION OF THE *E. coli* LAC OPERON BY THE CAP ACTIVATOR IN 1984

**FIGURE 1:**
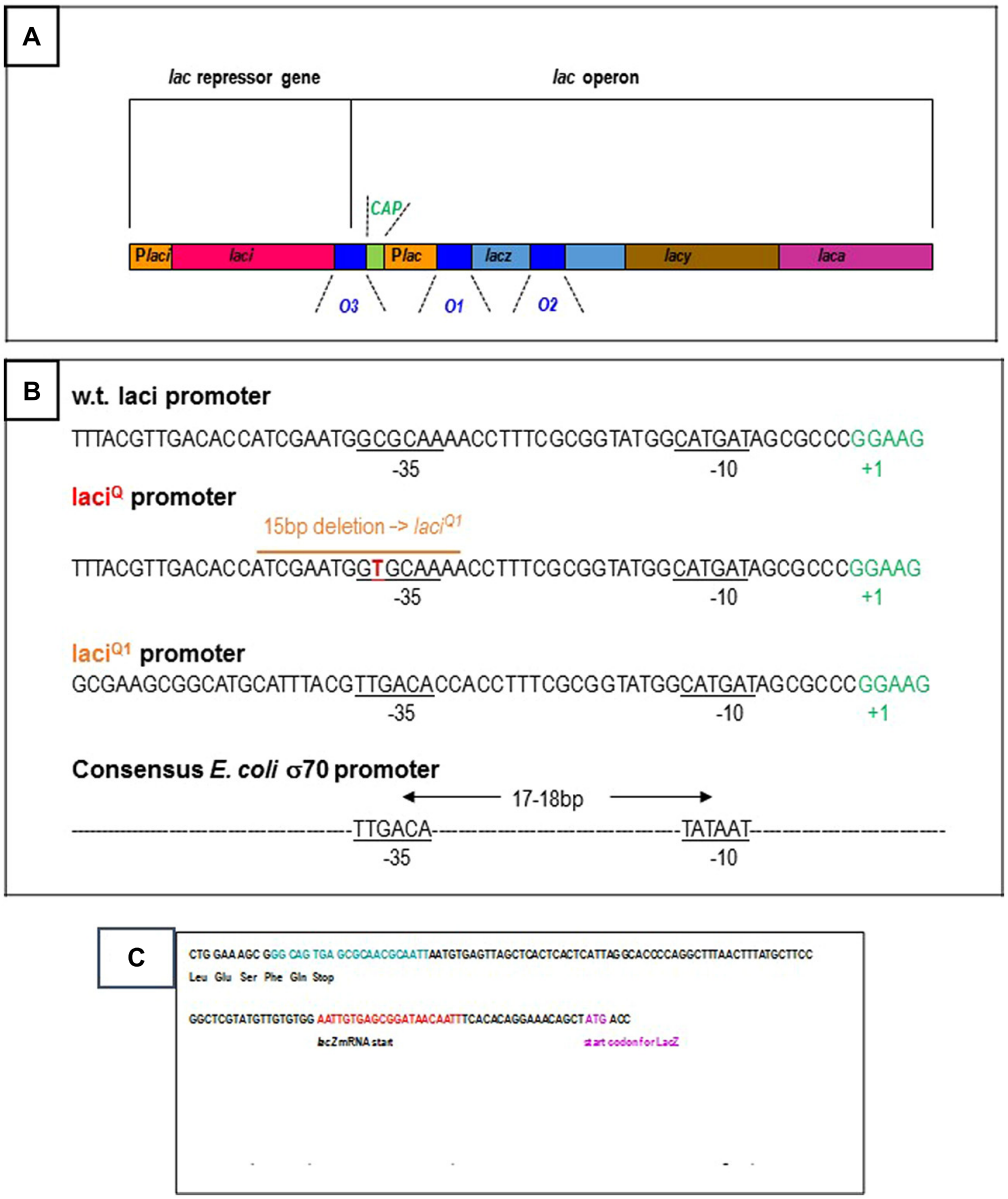
**(A)** Schematic organization of the *Escherichia coli* lac operon: the three structural genes, the beta-galactosidase (lacz, light blue bar), the permease (lacy, brown bar), the transacetylase (laca, pink bar) are controlled from the Plac promoter region (yellow bar). They are repressed by the lac repressor from three operators (dark blue bars): O1 is located on the promoter region and is a 21 bp DNA sequence centered at +11 with respect to the transcription start, O2 is located within the beta-galactosidase gene at +401, O3 is located upstream of the operon at -82. When required, the operon is activated by the CAP, green bar from the Plac promoter. The lac repressor is synthesized from the laci gene (red bar) and the Placi promoter (first yellow bar). The details are not drawn to scale. **(B)** 5′–3′ DNA sequence of the wild-type and over producing *E. coli* lac repressor genes: the promoter regions of the wild-type laci gene and of the mutated laci^Q^/laci^Q1^ genes are specified. Repressor production is, respectively, 10 and 100 times higher from laci^Q^ and laci^Q1^. The laci^Q1^ deletion creates an ideal -35 box (consensus sequence for the standard *E. coli* sigma-70 promoters: TTGACA) ideally located at 18 bp from the -10 box (consensus sequence: TATAAT). **(C)** 5′–3′ DNA sequence of the wild-type *E. coli* lac repressor gene: the codons for the five terminal amino-acids and stop codon are indicated. The O3 operator is located at the very end of the gene and is specified in blue. The beginning of the adjacent lac operon region is also specified, with the O1 operator (in red), the start of the beta-galactosidase mRNA transcript and that of its protein coding sequence.

Several basic features of gene regulation have been established in the Molecular Biology Department of the Pasteur Institute (Jacob et al., 1960). The *E. coli* lactose operon has provided a model for these studies. The three structural genes of the lac operon synthesize the enzymes in charge of lactose utilization in the bacterium (**Figure 1A**). The beta-galactosidase catalyses the breakdown of lactose into glucose and galactose. It also catalyses the isomerization of lactose into allolactose. The permease is needed for the transport of lactose from the growth medium to the cell. The transacetylase transfers an acetyl group to some beta-galactosides, a function which remains obscure for lactose catabolism. The three genes are expressed and controlled by the same elements from a unique promoter region. In the presence of glucose in the cell environment, the repressor protein acting from an operator site prevents gene expression. In 1984, only the O1 operator located on the promoter region is thought to be functional. The two other operators, O2 and O3, with sequence homology to O1, are regarded as evolution left-overs. Lactose is only degraded if glucose is lacking in the growth medium. In this case, lactose derivatives (allolactose or isopropyl-thio-beta-D-galactoside, IPTG) weaken repressor binding to DNA. The CAP is a sensor of the glucose level in the medium. When repression is removed, the expression of the three genes can be switched on by the standard *E. coli* RNA polymerase (sigma70-RNA polymerase) and the adjacent CAP activator from the lac operon promoter.

A remaining question in 1984 is the question of how RNA polymerase is precisely activated by the adjacent CAP activator at the lac promoter. For some groups, CAP contiguity provides direct and specific protein-protein contacts for RNA polymerase activation (Blazy et al., 1980). More frequently, these contacts are thought to be fortuitous and unspecific. (1) In fact, extensive CAP mutagenesis to disrupt any contact with RNA polymerase is unsuccessful. One has to wait for the ulterior finding that a unique contact between CAP and polymerase is incredibly responsible for activation (Irwin and Ptashne, 1987). (2) DNA is rightly considered to be rigid when the activator and polymerase are close to contiguity. Because two CAP molecules are often present at the *E. coli* promoters, two molecules might relay activation (Irwin and Ptashne, 1987). (3) CAP has been shown by Crothers’ group to bend DNA by the circular permutation assay of gel electrophoretic mobility (Wu and Crothers, 1984). This local and protein-induced curvature might indirectly increase the affinity of RNA polymerase for its promoter downstream (see also Miyada et al., 1984). (4) In case of the lac control region specifically, CAP is also supposed to act indirectly by preventing RNA polymerase from binding at an unproductive promoter which overlaps the productive one (see Amouyal and Buc, 1987 for example).

### DNA AS A MACROMOLECULE: BIOPHYSICAL PROPERTIES ISSUED FROM ITS CYCLIZATION

The CAP activator locally bends DNA, as evidenced by electrophoretic gel retardation assays with DNA fragments carrying the CAP site at different places (Wu and Crothers, 1984). To confirm this induced curvature, DNA cyclization experiments are planned with the same fragments in the presence of CAP (Crothers et al., 1992).

**FIGURE 2:**
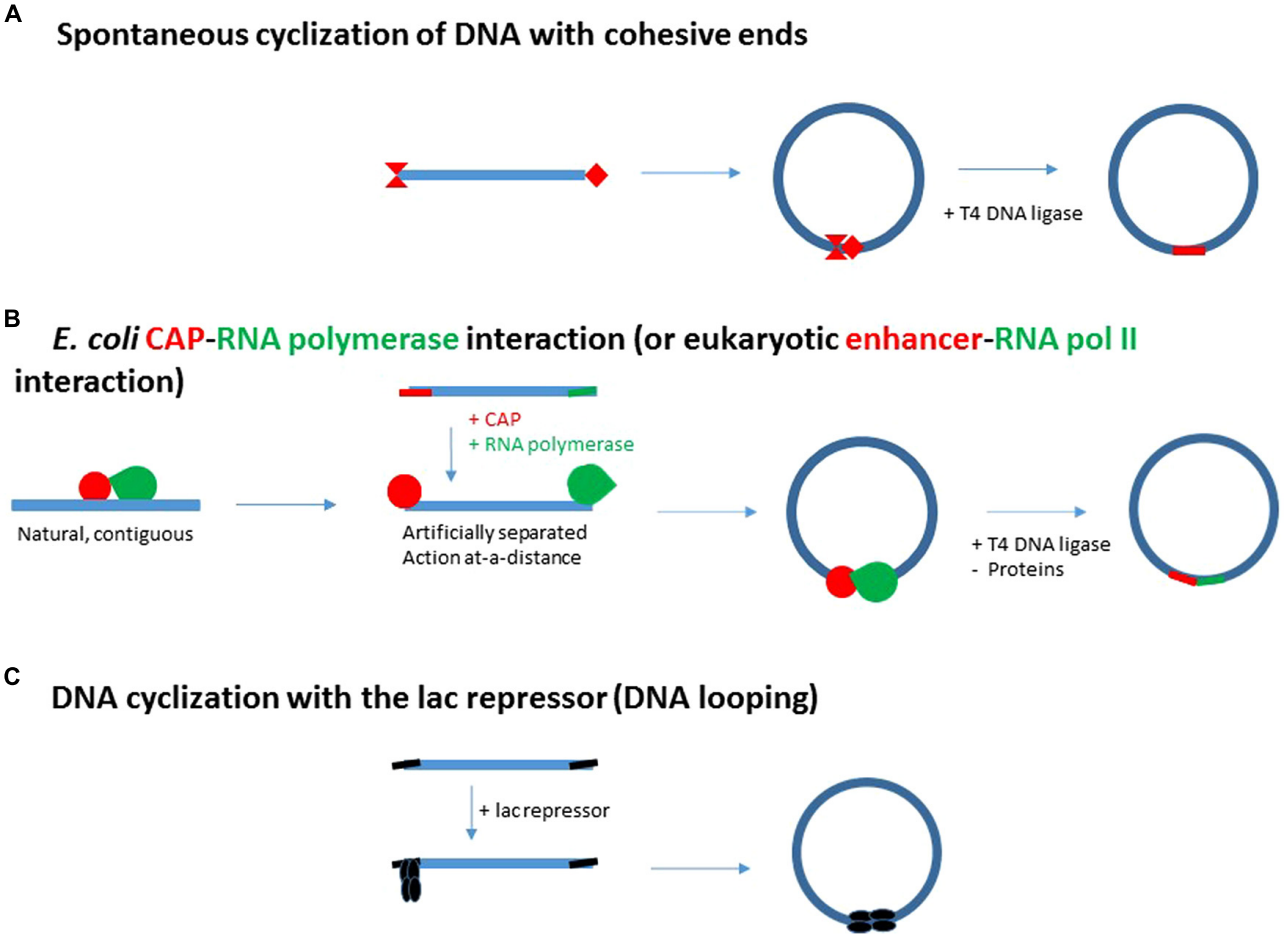
**From spontaneous DNA cyclization (A) and adjacent *E. coli* CAP-RNA polymerase interactions (B) to enhancer action (B) and DNA looping generated by the *E. coli* lac repressor (C). (A)** Schematic representation of the DNA cyclization assay for the determination of some specific properties of macromolecular DNA (bending flexibility, torsional rigidity and DNA persistence length). The ends of the fragment are cohesive and thus depicted by complementary red extremities. **(B)** Initial strategy to test the specific *E. coli* CAP activator – RNA polymerase interaction: the contiguous CAP site (in red) and promoter (in green) are artificially separated at each end of a DNA fragment. They then replace the cohesive ends of the DNA fragment in **(A)**. This step automatically turns adjacent interactions into distal, enhancer-type interactions. **(C)** The two distinct *E. coli* CAP and RNA polymerase proteins have been replaced by a single protein, the *E. coli* lac repressor. The *E. coli* lac repressor is a tetramer resulting from a dimer-dimer association (Kania and Müller-Hill, 1977). This tetramer can be hypothetically split up into a first dimer interacting with the promoter and a second dimer binding DNA distantly with the ability to enhance repression issued from the promoter. If the protein-protein and DNA-protein interactions generating the DNA loop, are stable enough, DNA ligation is not required to evidence DNA loop formation.

**FIGURE 3:**
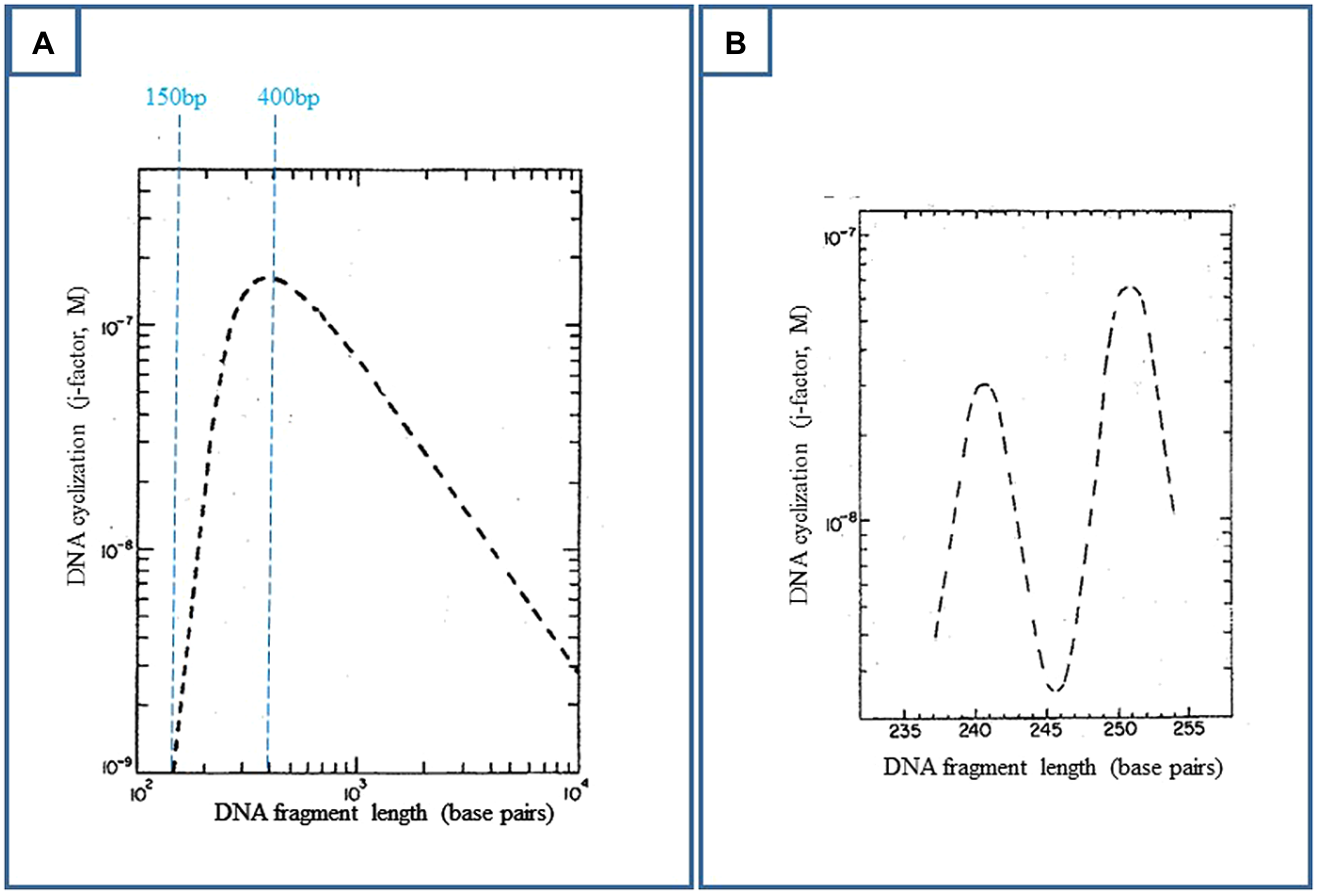
**Deoxyribonucleic acid ring closure probability (or j-factor) as a function of DNA length, when DNA is represented by a worm-like chain of segments with a persistence length of 150 bp (theoretical predictions).** Experimentally, DNA fragments with EcoRI cohesive ends and varying lengths are ligated by bacteriophage T4 DNA ligase. The percentage of covalently closed DNA with respect to remaining DNA is determined by electrophoretic gel retardation. This ratio reflects the DNA cyclization probability. The experimental data are in close agreement with the theoretical predictions (Shore and Baldwin, 1983; Horowitz and Wang, 1984, for example). **(A)** Adapted from Shore and Baldwin (1983); Ring closure is optimal for 400 bp. Above this value, though DNA keeps its bending flexibility, the two extremities of the fragment have less probability to find each other. **(B)** Adapted from Shore and Baldwin (1983); Below 400 bp, especially below 250 bp, DNA becomes torsionally stiff and the two cohesive ends must be brought into proper helical alignment (“in phase”) for covalent joining by the ligase.

Crothers’ planned study is based on various works between 1981 and 1984 dealing with spontaneous DNA cyclization (see for example Shore and Baldwin, 1983; Horowitz and Wang, 1984, **Figures 2A** and **3**). These studies have shown that as a large chain of polymerized nucleotides, DNA shares similar properties with its chemical analogs (Jacobsen and Stockmayer, 1950). In this respect, DNA is characterized by a bending flexibility, a torsional rigidity and a persistence length (**Figure 3**).

### DNA CYCLIZATION TO ASSAY THE DIRECT CAP-RNA POLYMERASE INTERACTION

In 1984, starting molecular biology (see short biography in supplementary material), I am immediately interested in proving a specific contact between RNA polymerase and CAP on the model of Crothers’ planned work with the sole CAP activator. If the contact for activation is specific instead of being incidental, DNA cyclization of a fragment carrying the CAP site and the promoter at each end of the fragment must be accelerated (**Figure 2B**).

On the other hand, due to DNA bending flexibility, the spontaneous cyclization of a DNA fragment is the most probable over the DNA persistence length, when its size lies between 150 and 2000 pb, and is optimal for 400 bp (**Figure 3A**, Shore and Baldwin, 1983; Horowitz and Wang, 1984 for example). The concentrations of DNA must be weak enough to prevent DNA aggregation. These conditions could also describe the best conditions for cyclization of a DNA fragment induced by RNA polymerase and CAP interaction, as probed by DNA ligation (**Figure 2B**).

In this light, the activation by CAP through local DNA bending is turned into an activation by a protein-protein contact, a naturally contiguous contact of activation is replaced by an artificially remote contact from two clearly separated CAP and polymerase sites (**Figure 2B**).

The physical interaction was later shown by means of these same DNA cyclization/ligation assays without separating the CAP and promoter sites and by placing them in the middle of the DNA fragment (Crothers et al., 1992), as well as genetically (Irwin and Ptashne, 1987). A genetic system was also later built to artificially generate activation by the CAP activator at some distance of a lac promoter and gene (work by Koop and Bourgeois, 1991, personal communication by S. Bourgeois). These constructs were not productive: CAP only activates *E. coli* RNA polymerase from its natural adjacent locations on the promoter. In fact, proximal activators are not suited for remote activation because they do not make the same contacts of activation with the transcriptional complex of initiation (Seipel et al., 1992). Thus, when *E. coli* cells are deprived of nitrogen, the paired NtrC activator is phosphorylated and RNA polymerase replaces the standard sigma-70 component by the sigma-54 component to make enhancer-like contacts.

### THE DISCOVERY OF TRANSCRIPTIONAL ENHANCERS IN EUKARYOTES IN 1981

In 1984, these elements have been newly unraveled by two groups (Banerji et al., 1981; Moreau et al., 1981). In other words, eukaryotic genes are found to be regulated by sequences and proteins located far from the gene on the chromosome. They activate gene expression independently of their orientation and distance with respect to the gene, which will become their definition (Yaniv, 1984).

### THE *E. coli* LAC REPRESSOR IS ABLE TO REPRESS EXPRESSION OF AN ARTIFICIAL SYSTEM

In 1985, another set of data broadens the prospect of the initial project. Muller-Hill’s group has just found that the lac repressor is able to repress beta-galactosidase from two synthetic operator sites which surround the promoter and are separated by 220 bp in an artificial system (see next section, Besse et al., 1986). The nucleoprotein complex which is formed between the fragment carrying the 220 bp-spaced operators and the lac repressor, remains to be identified.

### FROM ACTIVATION TO REPRESSION

This artificial lac repression system has several advantages. (1) The genetic constructs carry high affinity sites, the so-called “ideal” lac operators, which avoids T4 DNA ligation. (2) lac Repressor is made of the aggregation of two dimers. This permanent association may advantageously replace the transient protein–protein association of CAP and RNA polymerase in the assays (**Figure 2C**).

Gel retardation is the technique which is selected in the first place. The assays are expected to reveal an abnormal electrophoretic mobility of any species different from one operator simply bound to the repressor on the same fragment. Consequently, all species possibly formed between a fragment carrying two lac operators and the lac repressor are expected to be discriminated from this simple complex, as well as between them in the gel: repressor-induced DNA loops, simple repressor binding to one operator, tandem repressor binding to two operators, DNA aggregates as well.

Indeed, this very first step was sufficient to show that the lac repressor could easily induce DNA looping when the two operators were separated by distances close to the 150 bp DNA persistence length and up to 220 bp. This finding was made directly readable in electrophoretic gels by Krämer et al. (1986, 1987).

Nevertheless, enhancers function at much larger distances, whereas large loops are too much retarded in the gels to be easily detected by this technique. Electron microscopy (em) is in fact better suited for the observation of large loops. The optimal size of a DNA fragment for its spontaneous cyclization is 400 bp (**Figure 3A**). When applied to the genomic cyclization induced by the lac repressor, this property implies a 400 bp spacing between the two operator sites. In this case, em grids are quantitatively covered by a field of loops when the preparation is that of the gel retardation assays^∗^. The first tested spacing between operators was actually larger, 535 bp. This spacing was unproductive in the Besse et al. (1986) work *in vivo* because of concentration effects (next section), but is closer to the theoretical 400 bp value than the *in vivo* productive 220 bp distance. For this optimal spacing, as standard DNA spontaneously cyclizes, it is important to check for artifacts. For example, internal and spontaneous 400 bp coils are naturally observed on em grids within long DNA fragments in the absence of protein. In an ulterior set of assays, larger operator–operator distances were also successful, up to 2000 bp. em also confirmed the electrophoretic DNA loop formation below 220 bp.

This very first work with linear bacterial DNA (Krämer et al., 1986, 1987) and the studies that immediately followed, with natural operators as well as artificial ones, showed that a transcription factor (depicted by the *E. coli* lac repressor) could simultaneously interact with two distant regions responsible for gene regulation (depicted by the two *E. coli* lac operators), generating genome folding. This finding was reproducible, quantitative and consistent with the properties initially defining eukaryotic enhancers.

(1) Repressor-bridged genome looping is not incidental, nor punctual and specific of a given distance or DNA-protein arrangement with some degree of flexibility. Rather it is possible over a wide variety of spacings between operators (first property of eukaryotic enhancers).
(2) As predicted by the DNA cyclization model at large distances and experimented with several lac operators, DNA looping is consistent with either orientation of the genomic sites with respect to transcription (second property of eukaryotic enhancers).

The DNA cyclization model revealed a supplementary condition of feasibility which was not expected from the comparison with eukaryotic enhancers: the concentrations of transcription factor and genomic fragment had to be low enough *in vitro* to avoid saturating the genomic sites with protein or the formation of DNA-protein aggregates (Amouyal, 2007a).

^∗^The 400 bp optimal distance for enhancer-induced genomic cyclization was also used to evidence DNA looping by em with the mammalian estrogen receptors, as well as with the NtrC regulatory component of the *E. coli* ammonia deprivation system (Su et al., 1990), as both suggested by the author.

### THE BACKGROUND THAT THE BEGINNER IN BIOLOGY ONLY LEARNT AFTER COMPLETION OF THE INITIAL PROJECT

(1) A variant of the genomic folding hypothesis had been considered in a different context, that of specificity: how a transcription factor was able to distinguish a specific DNA sequence from its unspecific environment? Was the protein sliding along DNA until its specific site? Was it unspecifically jumping from place to place on the genome until reaching the specific target (“hopping”)? Was it transferred to the specific site by unspecific and transient “intramolecular transfer”? (Riggs et al., 1970). Several *in vitro* data had concluded to sliding of the lac repressor (see for example Winter et al., 1981).
  Transient intra-transfer was supported by the presence of two DNA binding sites on lac repressor (Kania and Müller-Hill, 1977; Culard and Maurizot, 1981). Kania and Müller-Hill (1977) had also obtained genetic data indicating that the second operator was weakly involved in beta-galactosidase repression. However, these data could not be reproduced by another group, preventing their publication (Müller-Hill, personal communication, 1987). Furthermore, several biophysical techniques and electron microscopy, including in Maurizot’s and Müller-Hill’s laboratories, had not detected such an intra-transfer of lac repressor. Consequently, involvement of the O2 and O3 operators to repression of the lac operon was excluded.
(2) In 1984, shortly after the discovery of eukaryotic enhancers, only the *E. coli* arabinose operon is clearly shown to be repressed *in vivo* from two clearly upstream operator sites separated by 210 bp (up to 240 bp artificially) with a requirement for phasing between the two distant operators *in vivo* (Dunn et al., 1984). The involvement of two operators to galactose repression by GalR (Majumdar and Adhya, 1984) is complicated by the presence of two productive promoters (Kuhnke et al., 1986). The Besse et al. (1986) work with the corresponding artificial lac system, which I only know and is only under way at that time (the reason why Müller-Hill contacts me in 1985), has been designed to mimic the genetic organization of the galactose operon and to support multi-operator repression of the gal operon, while this is excluded for the lac operon.
(3) While the Krämer et al. (1986) gel retardation data with distances from 220 bp down to the 150 bp DNA persistence length (**Figure 3**), are waiting for em and larger distances, Hochschild and Ptashne, 1986 show that the lambda cI dimer binds cooperatively to two contiguous sites. This cooperative process precludes the requirement for strong affinity sites, a key point for repression of the lac operon (see next section). The lac study that has started with distances superior to the DNA persistence length in gel retardation assays, is extended to distances close to contiguity reproducing the spacing between the lambda cI operators, down to 56 bp. In this case, DNA looping is partially or totally reduced to DNA coating by the protein (Krämer et al., 1987; Amouyal et al., 1998). Later on, with *in vivo* chemical probing, Borowiec et al., 1987 will show the surprising involvement of the lowest affinity O3 operator to lac repressor binding in cooperation with the O1 operator located in the promoter region.

## FURTHER DEVELOPMENTS

### REPRESSION OF THE *E. coli* LAC OPERON. HYPOTHETICAL AUTOREPRESSION OF THE REPRESSOR THROUGH O1–O3 GENOMIC CYCLIZATION

The *in vitro* studies had shown that repressor-induced DNA looping was favored by low concentrations of operator sites and repressor. Operator and repressor concentrations in the cell are also low. In the wild-type situation, there is only one copy of lac operon when it is carried by the chromosome and only 10 copies of lac repressor per cell. When the genetic studies were performed as close as possible to these natural conditions, the two operators O2 and O3 which are not located on the promoter, were found to strengthen repression issued from the proximal operator O1 by 72-fold. When strains overproducing the repressor were used, the contribution of the distant sites was nearly erased in the cells (Oehler et al., 1990, 1994; Amouyal and von Wilcken-Bergmann, 1992). These conditions were frequently used in the past with the rationale behind that in order to see the contribution of distant sites, one had to make sure that the repressor is effectively bound to the sites. However, these concentrations saturate the sites and preclude DNA looping. As a result, the contribution of the non-promoter lac operator sites O2 and O3 was skipped or underestimated (see Besse et al., 1986, for example). For technical reasons of commodity, multi-copy plasmids are also often used with the same risk of masking the contribution of distant sites to regulation.

With O3, the lac operon shares a common feature with eukaryotic gene regulation. The affinity of this sequence for the repressor is so weak that repressor binding cannot be detected by classical techniques *in vitro*. Though O3 had been shown to interact *in vivo*, it was not expected to be an auxiliary of repression (Borowiec et al., 1987). In fact, the contribution of O3 to beta-galactosidase repression is far from being modest. O3 contributes to repression through DNA looping. (1) Repressor binding *in vivo* only occurs in the presence of the proximal operator O1 (Borowiec et al., 1987). (2) A dimeric repressor unable to tetramerize has the same effect as a tetrameric repressor at O1 alone (Oehler et al., 1990). (3) A high level of cooperative repression is obtained from two O3 or even weaker sites (Amouyal and von Wilcken-Bergmann, 1992; Oehler et al., 1994). (4) The CAP activator, absent from some artificial constructs, cannot be responsible for this repression by interacting with repressor at (or near) O3 (Amouyal and von Wilcken-Bergmann, 1992; Perros and Steitz, 1996 and the whole discussion related to repressor-operator crystallization in Science 1996). (4) Repressor sliding along genomic DNA is excluded (Amouyal and von Wilcken-Bergmann, 1992).

In the wild-type situation, O3 is nearly as efficient as O2 in strengthening O1 repression (25-fold for O3, 40-fold for O2, Oehler et al., 1990). In an artificial system and in cooperation with itself (two O3 operators), O3 is able to repress beta-galactosidase as efficiently as O1, up to 35-fold (Amouyal and von Wilcken-Bergmann, 1992). So that whether O1 interacts with O3, or with O2, beta-galactosidase is very efficiently repressed through DNA looping (440 and 700-fold, respectively, Oehler et al., 1990).

If the strong operator O1 replaces the weakest operators O2 or O3, repression is even more efficient, but inducibility is totally or partially lost (Amouyal and von Wilcken-Bergmann, 1992; Oehler et al., 1994). The level of repression has to be determined by comparison with an identical strain which lacks the repressor, instead of the inducer. Modulation of DNA binding by gene-specific transcription factors is a crucial element and some aspects still have to be cleared (Schleif, 2013). Replacing the weak operators by the strong one has qualitatively the same effect (high level of repression) as replacing the repressor by a mutant superrepressor which binds tightly DNA (Willson et al., 1964; Bourgeois and Jobe, 1970). Yet, these superrepressors have not been naturally selected, nor a set of strong operators. Instead, a system of weak operators has been naturally selected. Then, if DNA looping is necessary to have efficient repression, low affinity sites are required to keep the operon inducible.

In the wild-type situation, alternative DNA looping involving either the O1/O2 or O1/O3 pair of operators, increases the probability of obtaining a high level of repression from the proximal operator O1 in each cell (Oehler et al., 1990). However, if the O1–O2 loop strengthens repression at the promoter, it also consolidates beta-galactosidase repression from O2 by blocking any RNA elongation at O2 (Flashner and Gralla, 1988). DNA loop formation is required for this process, since O2 alone is unable to stop RNA elongation. The O1–O3 loop may play a similar role.

The O3 operator is located at the extreme end of the laci gene (**Figures 1A,C**). The view that the laci gene is not regulated, is anterior to the finding that O3, in spite of its low affinity for the repressor, is involved in repression of the lac operon. However, the O1–O3 loop might contribute to self-repression of the repressor simultaneously to repression of the operon. This would not be surprising since several *E. coli* repressors are autorepressed, as reviewed by Collado-Vides et al. (1991). Furthermore, Selliti et al. (1987) have noticed that several transcripts are synthesized from the laci gene and that RNA elongation is blocked within the control region of the lac operon, or before. In particular, one of these transcripts ends 5 bp upstream of the lacO3 operator, within the laci gene and before the lac control region, in the presence of repressor. This truncated transcript is less stable than the transcript giving rise to the lac repressor, according to Selliti et al. (1987).

If the repressor simultaneously bound to O3 and O1 (since O3 alone is unable to do so) stops RNA elongation, like O2 through O1–O2 DNA looping, this would not be the main process of limiting lac repressor production. This production is already limited, at the overall level of 10 molecules per cell, by a weak promoter (Müller-Hill et al., 1968; **Figure 1B**). This is confirmed by mutations in the promoter region that increase repressor production. For example, a particular laci^Q^ mutant produces about 10-fold more repressor (Müller-Hill et al., 1968) thanks to a single CG →TA base pair mutation located at -35 of the laci promoter (Calos, 1978). A 15 bp deletion in the promoter region leads to a 50–100-fold increase of lac repressor synthesis, creating an improved –35 homology region and a strong promoter (Calos and Miller, 1981).

Another element has to be taken into account: the gene producing the repressor is tied to the lac operon (**Figure 1A**). In most other *E. coli* operons, the gal operon or the deo operon for example, respectively, responsible for galactose and for deoxynucleoside catabolism, the repressor is not produced in close proximity to the promoter. Thus the apparent concentration of lac repressor available for gene regulation is higher than the 10 molecules actually measured. In this context, if a weak promoter is necessary to produce the first molecules required for repression and to limit this production, the O1–O3 loop is necessary to stop it after the initial burst of production.

### THE ROLE OF GENOMIC STRUCTURE AND CONFORMATION IN GENOMIC CYCLIZATION AND GENE REGULATION

#### DNA supercoiling in *E. coli* facilitates the folding of the genome

Deoxyribonucleic acid is supercoiled in bacteria. DNA superhelicity is half that found for plasmids extracted from bacteria (superhelix density sigma = -0.06, Buc and Amouyal, 1992 for example). This superhelicity facilitates DNA looping (Borowiec et al., 1987; Krämer et al., 1988). Thus, *in vivo*, the lac O3 operator only interacts with its site in the presence of DNA supercoiling (Borowiec et al., 1987). With mini-circles of 452 bp carrying 153–168 bp distant lac operators possibly forming loops, i.e., when the spacing between operators is close to the DNA persistence length (**Figure 3A**), it becomes possible to measure DNA torsional rigidity and helical pitch. DNA supercoiling modifies this pitch and the subsequent mutual orientation of two sites of regulation. Thus *in vivo* the helical pitch might be different from the 10.5 helical pitch measured on a DNA fragment (Bellomy et al., 1988; Krämer et al., 1988; Lee and Schleif, 1989; Perez et al., 2000).

As a secondary set of data, it was possible to experimentally check a mathematical theory about mini-circles. This theory predicts that under a moderate constraint, small circles remain flat and toroid instead of being writhed (Le Bret, 1979). It was verified both by electron microscopy and through DNA looping with lac repressor. The method which has been used, leads in principle to the distribution between the torsional stress and the writhing stress in plasmids with a known superhelical density.

#### The role of enhancer orientation

Eukaryotic enhancers act independently of their orientation with respect to the start of transcription. This is consistent with DNA looping at large distances. In this case, the flexibility of genomic DNA allows various conformations of the loop. However, DNA looping might become sensitive to enhancer orientation at short or moderate distance (Amouyal, 1991). Even in this case, the protein (or protein complex) involved in DNA looping has to be asymmetric, as are heterodimers (Amouyal, 1991; Zhou et al., 1993) or insulator complexes (Kyrchanova et al., 2008). If the protein is symmetric, like lac repressor, in order to detect a differential effect of enhancer orientation with respect to short and large distances, the asymmetry of the binding site has to be transduced to the protein (Amouyal, 1991). Beta-galactosidase expression does not depend on the mutual orientation of two asymmetric operators at short distance (the O1–O3 spacing), revealing some protein flexibility (Amouyal and von Wilcken-Bergmann, 1992). NMR confirms that protein plasticity erases the asymmetry of the repressor–operator interface (Kalodimos et al., 2002).

#### Genomic cyclization is facilitated by histones in eukaryotes: the role of histones in providing a long-distance action

The debate about the mode of action of enhancers in the Pasteur Institute (Yaniv, 1984) had locally prompted the Krämer et al. (1986, 1987) study as an answer.

In 1990, several *E. coli* operons have been found naturally regulated in a way reminiscent of eukaryotic enhancer action. They are actually repressed, from operators located outside of the genes like eukaryotic enhancers, and at least outside of the promoter region. However, five major criticisms prevent to directly apply the prokaryotic DNA looping model to eukaryotes.

(1) Natural distances of action are still of the order of a few 100s of base pairs in prokaryotes, instead of tens of thousands of base pairs in eukaryotes. In the *E. coli* deo operon, DNA looping enhances repression up to 900 bp naturally and up to 5000 bp artificially *in vivo* (Dandanell et al., 1987; Amouyal et al., 1989). Nevertheless, the prokaryotic generators of distant effects could not (and were not) classified as “transcriptional enhancers”.
(2) In the wild-type situation, *E. coli* analogs of enhancers are only observed for repression instead of activation. Remote activation in *E. coli* is only artificial, issued from the genetic constructs related to nitrogen deprivation and does not exceed 1000 bp (Reitzer and Magasanik, 1986). From this point of view, *E. coli* has not come closer to eukaryotes. On the contrary, eukaryotes have come closer to prokaryotes with the so-called silencers in yeast which can repress gene expression remotely in either orientation (Brand et al., 1985).
(3) The eukaryotic components of regulation are more complex than their prokaryotic counterparts, with different partners and chromatin actors absent from bacteria. The complexity of prokaryotic components has somewhat evolved with the finding that protein aggregation often goes with long distance action in *E. coli*.
(4) Enhancer action could not be reproduced *in vitro* in eukaryotes (Müller-Storm et al., 1989).

A first step for a common basis of enhancer action in prokaryotes and eukaryotes has consisted in clarifying the role of histones. When dealing with eukaryotic enhancers, the distance of action is always indicated by the number of base pairs between the enhancer and the promoter, for example 10000 bp. However, if the enhancer acts through chromatin cyclization, the distance of action is shorter. In this case, DNA cyclization applies to chromatin instead of simple DNA. If DNA is compacted x-fold around histones, the enhancer distance of action is also reduced, from 10000 to 10000 bp/x in the given example. This reduces the discrepancy between prokaryotes and eukaryotes concerning the enhancer distance of action.

In fact, the use of nuclear extracts containing histones allowed to reproduce enhancer action *in vitro* (Seipel et al., 1992), contrary to previous work (Müller-Storm et al., 1989). Thus, histones bring enhancer closer to the gene by compacting DNA and facilitating chromatin cyclization (see next section). Doing so, they passively activate expression. At that time, they were only assumed to be responsible for a repressive effect, when heterochromatin coats the gene.

Furthermore, in this context, having lac repressor operate at-a-distance from a gene in eukaryotes should give an idea of DNA compaction in nucleosomes (Amouyal, 1991). Lac repressor has been known for long now to function in eukaryotic cells (Hu and Davidson, 1987). Ideally, the same transcriptional machinery (promoter and RNA polymerase) would also be required in *E. coli* and the selected eukaryotic cells in order to rigorously compare the distance of action of the repressor in both bacterial and eukaryotic cells. This is in principle possible since some bacterial RNA polymerases, the phage T3 and T7 RNA polymerases, can also operate in eukaryotic cells (Deuschle et al., 1989).

More simply, some chromatin regions of the eukaryotic genome carry labile variants of standard histones, H3.3 or H2A.Z (see Amouyal, 2010b) or are devoid of histones like in prokaryotes (Kleinschmidt et al., 2011). Comparing the action of an artificial DNA looping device between these regions and standard chromatin in the same cells or organism, might give some information on the chromatin state in these regions.

A first step in this direction was a study suggested by J. F. Nicolas, head of the Developmental Biology section of the Pasteur Institute. This preliminary work in NIH3T3 cells (Amouyal, 1996) intended to introduce a possible animal binary system for cell fate analysis and developmental studies (Byrne and Ruddle, 1989; Petit et al., 2005). These binary systems usually consist of a mute mouse strain carrying a construct unable to express a given gene, and of an activating mouse strain bringing the transactivating component when the two strains are crossed. In this particular case, the gene was a reporter gene under the control of the T3 promoter. The activator is the T3 RNA polymerase itself under the control of a promoter of interest, so that the T3 promoter and the T3 RNA polymerase are directly the induced and inducing components. The promoter of interest is switched on at a precise step of the development and in a defined tissue, which allows to follow its activity in a given cell line.

#### Genomic cyclization is facilitated by histones in eukaryotes: *in vitro* verification and application to the synthesis of large recombinant DNA

In the previous section, it was assumed that histones facilitate DNA ring closure. This can be simply checked by DNA ligation in the presence of histones. As bacteriophage T4 DNA ligase is the ligase used for the molecular cloning of fragments into plasmids, verifying this hypothesis naturally evolves as an application of molecular cloning. In this case, histones (a mixture of commercialized histones from calf thymus) are involved at the level of ligation of the linearized plasmid vector with insertion fragment. As a result of improved cyclization, an increased number of transformants is obtained when *E. coli* is transformed with this mixture (Amouyal CNRS Patent 1999–2002, Amouyal, 2007b). This is also simply observed with the sole linearized vector in the absence of insertion.

The 3C technique (Dekker et al., 2002), which allows to draw a systematic picture of protein-protein interactions involved in distant chromatin folding in eukaryotic cells, relies on a somewhat similar basis.

### THE BRIDGING PROTEIN: THE EFFECT OF ITS STRUCTURE ON GENOMIC CYCLIZATION AND GENE REGULATION

#### Genomic cyclization is possible with weak-affinity DNA-protein or protein-protein interactions, like those found in eukaryotes: application to their detection.

The DNA cyclization model introduced in the present synthesis relied on the need to probe the physical interaction between two proteins, the *E. coli* CAP and RNA polymerase.

In case of the tetrameric lac repressor, this protein-protein interaction is an internal part of the repressor (in fact a dimer-dimer association, Kania and Müller-Hill, 1977, **Figure 2C**). The arabinose repressor, AraC, is a monomer-monomer association (Frato and Schleif, 2009). The deoR repressor is made of 3, maybe four dimers (Amouyal et al., 1989). The phage 186 cI repressor includes seven dimers (Wang et al., 2013). On the contrary, for some other *E. coli* repressors, this interaction is provided by two separate molecules.

Thus, the gal operon is responsible for catabolism and uptake of galactose in *E. coli*. The operon may be repressed by the GalR repressor when it resides on either side of two close transcription starts, with a short 11 4bp distance separating the two operators (Majumdar and Adhya, 1984). The interaction between the two GalR dimers which could not be detected *in vitro* because it is too weak, is evidenced by beta-galactosidase repression in simplified genetic constructs reproducing the lac O3–O1/promoter organization (Perez et al., 2000).

The Perez et al. (2000) genetic system was initially aimed at determining the yeast RAP1- RAP1 interaction in *E. coli*, in a collaboration proposed to Suzanne Gasser and Eric Gilson in 1988 in Lausanne (Switzerland). When this property is systematically applied to a whole genomic library in yeast, this leads to the so-called “double hybrid” technique. This method allows to isolate all proteins interacting with a given one at short distance, whether this one is from yeast or from another eukaryotic organism (Fields and Song, 1989).

Another technique has been developed more recently to detect the internal interaction between the two monomers of the arabinose repressor (Frato and Schleif, 2009). The protein is so poorly soluble and this interaction so weak that it had never been detected by conventional techniques. The Frato and Schleif (2009) technique takes profit of an oligonucleotide flexibly and covalently attached to the protein. The dimerization of the protein facilitates the pairing of the complementary oligonucleotide with fluorescence quenching. Like the lac repressor- 03 interaction or the GalR-GalR interaction, AraC monomer-monomer interaction is reminiscent of eukaryotic interactions, such as those involved between the various factors of a transcriptional enhancer complex.

#### High-order protein complexes favor enhancer action

Eukaryotic enhancers are characterized by their large distance of action. DNA compaction around histones provides one explanation for the observed discrepancy between prokaryotes and eukaryotes. Another difference comes from the structure of the protein complex at the base of the loop. Large aggregates between multiple factors interacting with low affinity, including CTCF and cohesin for example, are generally observed in eukaryotes (see for example the Amouyal et al., 2011 on Gene insulation). Large protein complexes are more frequent than usually thought in prokaryotes, though the protein is simply aggregated. As underlined in previous section, the lac repressor is a 4-mer in the solution, the deoR repressor, a 6(maybe 8)-mer, the phage 186 cI repressor, a 14-mer. The dimeric phage lambda cI repressor octamerizes as an 8-mer through DNA binding (Pinkett et al., 2006). The NtrC activator acts as a pair of dimers. In all these cases, clear distance effects are observed and seem to be associated with the capacity of protein aggregation (Reitzer and Magasanik, 1986; Dandanell et al., 1987; Oehler et al., 1994; Pinkett et al., 2006).

Thus, the λ cI repressor is responsible for maintenance of the lysogenic state of integration of the phage lambda into *E. coli* genome and repression of the lytic process. In the wild-type situation, the lambda cI dimers bind DNA contiguously and cooperatively interact side by side. This protein arrangement is best adapted to the natural tandem organization of the genomic sites (Amouyal et al., 1998). Two dimers can be slightly separated on DNA and still cooperatively interact at distances which remain close to contiguity (Hochschild and Ptashne, 1986). Incidentally, if two sites are too close, the DNAse I footprints resulting from DNA coating by the protein and DNA cyclization are very similar (Amouyal et al., 1998).

On the contrary, when the dimers are not artificially separated and when the protein can both aggregate head to head (Perez et al., 2000), in addition to side by side (Amouyal et al., 1998), protein aggregation opens the way to long distance effects. In fact, the lysogenic process for the phage lambda cI repressor or its phage 186 cI analog only depends on long distance interactions if the phage cI repressor aggregates into a 8-mer or a 14-mer, like in the wild-type situation (Pinkett et al., 2006). Aggregation of the protein also allows the phage lambda cI repressor to occupy and to strictly repress its own promoter and its own production. This self-repression, resulting from protein aggregation, is crucial for an efficient switch from the dormant to the active state of the virus. If one of the operator required for lysogeny and lambda cI repressor self-repression is mutagenized, the concentration of lambda cI repressor is twofold to threefold increased with respect to the self-repressed state, counteracting the production of the Cro protein necessary for lysis.

Additionally, the natural O1–O2 spacing (390 bp) is close to the theoretical and experimental value of 400 bp found optimal for spontaneous *in vitro* DNA ring closure or DNA looping. All the works which have tackled the question of how far a repressor can repress at a distance from a first operator located on the promoter in *E. coli* have found that the level of repression was high from 200 to 500 bp (when DNA flexibility prevails on protein size and flexibility) and that it was decreasing afterward being still perceptible at 10 kbp depending on the protein (Dandanell et al., 1987; Oehler et al., 1994; Priest et al., 2014). Thus *in vivo*, the effect of distance on repression enhancement in *E. coli* roughly approximates the theoretical curves of DNA cyclization (**Figure 3**), confirming that the bacterial chromosome is not constrained by proteins like eukaryotic DNA by histones, and that it is not highly supercoiled.

#### The cellular level of transcription factors: new consequences on gene regulation and cell fate

In the first times of molecular biology, the urgency lied in isolating and characterizing the factors responsible for gene regulation. Therefore only their absence or presence, sometimes specifying the amount of the transcription factor in the cell, was noted. To analyze gene regulation, multi-copy plasmids and strains overproducing transcription factors were indiscriminately used. However, modifying the cellular level of transcription factors or of gene regulation sequences with respect to the wild-type situation, might mask a process of regulation. This point was crucial for DNA loop formation and repression of the lac operon, as detailed in the previous sections.

Most transcription factors are produced in the cell in a limited amount, because gene regulation, as an enzymatic process, only requires few molecules. Autorepression is a common way to restrain this cellular level. The TF6C subunit of the eukaryotic TFIIIC transcription factor is required by RNA polymerase III for the synthesis of transfer RNA. Recently, TF6C was found to be self-repressed when transcribed by RNA polymerase II, just like most prokaryotic repressors (Kleinschmidt et al., 2011). As TFIIIC is both a component of the machinery of RNA polymerase III transcription initiation and of genomic organization through the extra-TFIIIC elements (ETC sites, TFIIIC bound sites devoid of RNA polymerase III), the frontier between prokaryotes and eukaryotes, is like for enhancers thinner than initially thought.

In 1988, the finding that a natural gradient of bicoid protein determines the anterior or posterior position in the Drosophila embryo (Driever and Nusslein-Volhard, 1988) was the first example that protein concentration might determine cell fate. More recently, while pointing the role of concentrations on gene regulation (Amouyal, 2007a), a fruitful illustration of this focus was brought by Yamanaka and Takahashi (2006) who found that the concentration of only four key transcription factors controls the reversible transition from differentiated somatic cells to pluripotent stem cells.

The concentration of another protein, the Methyl-CpG binding protein-2 (MeCP2), is dramatically increased in neurons, pointing to a different role of the protein according to the type of cell (see for example Della Ragione et al., 2012 for a review). In neurons, MeCP2 concentration counter-balances H1 concentration, indicating that it could be involved in neuronal structure and plasticity via chromatin plasticity.

Though the concentration of these proteins is increased in a specific category of cells, they are produced in a limited amount.

This point can be developed further in a new way. In principle, overproducing a specific protein should be harmful to the cell. In the same line of ideas, simply delivering unspecific DNA and have it continuously transcribed or expressed in the cell should be harmful to the cell. This might underlie any therapeutic process requiring the killing of specific cells, such as cancer cells. However, in this case, it is generally important to target the unhealthy cells with both a process which is specific to cancer cells and a delivery which is also specific to these cells to avoid the risk of contaminating healthy cells. So that unless overexpression can be restricted to a definite category of cells (by a process similar to that described in Amouyal, 1996 for example), the lack of specificity of the strategy might limit the process.

However, the same strategy might be applied to close issues where the undesired cells can be differentiated from the endogenous ones for their targeting. This is the case of some pathogenic bacteria and of their resistance to antibiotics.

It should be noted that overproduction of a protein from a given DNA (or any related compound issued from this DNA) is not systematically harmful to the cell. Thus, ribosomal proteins or histones are produced in large quantities in all cells. Thus other elements would have to be considered, such as protein (or RNA) degradation for example.

### GENOMIC CYCLIZATION AND GENE INSULATION

Genes inserted into host genomes are deregulated, are not expressed in the desired cell type, or can deregulate host genes because of chromosomal position and undesired enhancer activity. Some sequences and proteins seem to partially protect them, naturally or artificially, from the neighboring environment. This is the case for the HS4 sequences, CTCF and cohesin proteins of the chicken beta-globin locus or of the scs/scs’ sequences of the heat-shock HSP70 locus in Drosophila.

The HS4 or scs/scs’ sequences delimit a transcriptional unit on the chromosome. Two operator sequences also surround the lac repressor-operator DNA loop and delimit a topological domain (Krämer et al., 1988). By analogy, the lac operator sequences might also act as gene insulators through DNA looping in eukaryotic cells producing the lac repressor (D. Bienvenu, B. Pineau and M. Amouyal, unpublished results, 1999–2000; Amouyal, 2010b). This was a way to postulate that gene insulators also operate through genomic looping. It now appears that insulators often provide an architectural prop for enhancer action through DNA looping when they do not simultaneously possess the capacity for both DNA looping and the enzymatic enhancer activity (Mongelard and Corces, 2001; Amouyal, 2010a,b, Amouyal et al., 2011 on Gene insulation, Amouyal et al., 2013). In some instances, they also stop RNA elongation, indirectly blocking heterochromatin propagation, like DNA looping to which it can be associated. Again, this dual mechanism is initially a property of *E. coli* transcription factors involved in DNA looping, such as the *E. coli* lac repressor.

## Conflict of Interest Statement

The author declares that the research was conducted in the absence of any commercial or financial relationships that could be construed as a potential conflict of interest.

## ABBREVIATIONS

AraC: arabinose operon repressor
CAP: catabolite gene activator protein
CTCF: CCCTC-binding factor
deoR: deo operon repressor
GalR: galactose operon repressor
NtrC: nitogen regulatory protein.
