# Supplementary material for: From adjacent activation in Escherichia coli and DNA cyclization to eukaryotic enhancers: the elements of a puzzle
Source: Front Genet. 2014 Nov 3;5:371. doi: 10.3389/fgene.2014.00371 (PMC4217526; doi:10.3389/fgene.2014.00371)
Supplement: Supplementary file 1 [file Presentation_1.PDF]

*Supplementary Material*

# From adjacent activation in E. coli and DNA cyclization to eukaryotic enhancers: the elements of a puzzle.

**Author :** Michèle Amouyal*

Interactions à Distance, Centre National de la Recherche Scientifique, Paris, France

*Correspondence: michele.amouyal@club.fr

## Short biography of the author

Michèle AMOUYAL has graduated as a chemical engineer from ENSIC (Nancy, France) and has been a researcher at the CNRS since 1975. She is a chemist until 1984. After a Ph. D. in macromolecular chemistry, she is in charge of a small group dedicated to N-carboxyanhydrides of alpha-aminoacids in Professor Sekiguchi's laboratory. In 1984, she joins the Molecular Biology Department of the Pasteur Institute (Paris, France) to define a biology-oriented interface of research (the CNRS has recently adopted "Surpassing the frontiers" as a motto). She is naturally led to tackle gene regulation in this location and decides that she will not return to Chemistry. In parallel to a work with Professor Buc on DNA supercoiling, she immediately starts in 1984 a personal project based on the DNA looping model in gene regulation, first in vitro and in E. coli in the Molecular Biology section of the Pasteur Institute. In order to specify this model in eukaryotes, she moves to the Cochin Institute of Molecular Genetics (ICGM, INSERM, Paris), then to the Developmental Biology section of the Pasteur Institute from 1992 to 1996 (Professor Nicolas). The initial phase in Biology (1984-1996) involves the non-academic supervising of different projects. She is finally appointed to Institut Gustave Roussy (Villejuif) and in charge of the "Interactions à Distance" group in 1997. There, she carries on her project in a pharmacological and biomedical background (UMR CNRS 147/1772/8532). Her researches follow these different orientations and environments.
